# Internet search data with spatiotemporal analysis in infectious disease surveillance: Challenges and perspectives

**DOI:** 10.3389/fpubh.2022.958835

**Published:** 2022-12-05

**Authors:** Hua Sun, Yuzhou Zhang, Guang Gao, Dun Wu

**Affiliations:** ^1^Popsmart Technology (Zhejiang) Co., Ltd, Ningbo, China; ^2^College of Computer Science and Technology, Zhejiang University, Hangzhou, China

**Keywords:** internet search data, spatiotemporal analysis, infectious disease, surveillance, prediction

## Abstract

With the rapid development of the internet, the application of internet search data has been seen as a novel data source to offer timely infectious disease surveillance intelligence. Moreover, the advancements in internet search data, which include rich information at both space and time scales, enable investigators to sufficiently consider the spatiotemporal uncertainty, which can benefit researchers to better monitor infectious diseases and epidemics. In the present study, we present the necessary groundwork and critical appraisal of the use of internet search data and spatiotemporal analysis approaches in infectious disease surveillance by updating the current stage of knowledge on them. The study also provides future directions for researchers to investigate the combination of internet search data with the spatiotemporal analysis in infectious disease surveillance. Internet search data demonstrate a promising potential to offer timely epidemic intelligence, which can be seen as the prerequisite for improving infectious disease surveillance.

## Introduction

In recent years, it has been realized that internet search data have great potential in infectious disease surveillance, which can be proved by increasing the use of such data to conduct rapid epidemics tracking and surveillance (1). As those data can offer timely surveillance intelligence with a high spatial resolution (2), they could be seen as a novel data source to monitor diseases and epidemics at both space and time scales.

The query “internet search data” in disease surveillance refers to social media data, internet search data, medicine sales data, and online news data (3). Infectious diseases continue to pose public health threats with a large social burden (4), and sometimes, they can cause significant pandemics, such as the coronavirus pandemic. To decrease the effects of infectious diseases on our society, it is critical to improve infectious disease surveillance (5).

The main aim of developing a disease surveillance system was to successfully predict possible diseases and epidemics or even outbreaks *via* their ability of early warning based on the data sources (6). Traditional infectious disease surveillance is based on the passive report system, which collects disease notifications from healthcare organizations. This kind of system is typically accurate but can delay up to 2 weeks from patients' diagnosis to the notifications being compiled into the surveillance system (7). As a result, this lag in the reporting process can post an adverse impact on the capability of the infectious disease surveillance system. Such a system may not offer real-time epidemiological intelligence, which leads to the reduction of the efficiency of infectious disease's quick response (8).

Public health experts consider spatiotemporal uncertainty in several manners. The geocoding process, e.g., using disease surveillance data with unreliable coordinates, could lead to spatial uncertainty. In addition, temporal uncertainty in disease surveillance typically causes a time lag between the occurrence of symptoms and case reporting *via* case identification after medical diagnosis (9). In the disease surveillance field, spatiotemporal uncertainty can present at the stages of data collection and statistical analysis. As a result, considering the spatiotemporal uncertainty can indeed contribute to the improvement of surveillance and even decision-making. The lack of spatiotemporal information in data collection and statistical analysis processes may lead to potential errors (e.g., false alarms) in disease surveillance and weaken the benefits of health actions, which is aimed to reduce the impact of diseases (10, 11).

However, few studies argued the use of internet search data with spatiotemporal analysis approaches in infectious disease surveillance. In this review, we present the challenges and possible future directions for researchers to investigate the combination of internet search data with spatiotemporal analysis in infectious disease surveillance.

## The application of internet search data

### The development of internet-based surveillance

Earlier, internet-based surveillance relied on online news as the main kind of data source, which results from the passive reading of online information for internet users at Web 1.0 (12). However, current studies of internet-based infectious disease surveillance use various internet data sources, including search query data from online search engines and social media such as Twitter (13). This mainly results from the information revolution and the rise of Web 2.0, which triggered the use of the internet as a new tool to actively and frequently seek health-related information (14). Thus, disease activity can be estimated by collecting and tracking changes in frequencies of related internet searches for key terms (15).

Several famous internet-based infectious disease surveillance systems have been successfully built using non-structured, event-trigged, internet search data. The Public Health Agency of Canada developed The Global Public Health Intelligence Network (GPHIN) to assist public health agencies, as well as the World Health Organization (WHO) Global Outbreak Alert and Response Network to detect infectious disease outbreaks using retrieved online information, such as online news. The network has first displayed its great ability during the severe acute respiratory syndrome (SARS) outbreak in 2003, with 2-months earlier reports for SARS than the official one by WHO (16).

Moreover, in the age of “Web 2.0,” the technologies of the proliferation of Really Simple Syndication (RSS) and Asynchronous JavaScript and XML enable researchers to develop more interactive infectious disease surveillance, such as HealthMap (17). This powerful internet search data-based surveillance used a wide range of internet external feeds, such as online news to collect valuable disease-related information, and then visualized the critical information, such as disease type, date, and location to the public as an early warning.

### The internet search data type

A variety of internet search data can be applied for infectious disease surveillance. Generally, the applied categories include internet search metrics (The volume of internet search activity) and mined social media data (The volume of social media posts). Additionally, the combination of the above internet data source with other data sources also has a great potential for surveillance, such as self-diagnosis questionnaires online, medication sales data, and school absenteeism data.

As a new tool, internet search data relies on the basis that the population group who have a great possibility of infections will actively search related information online about their health conditions. Thus, disease epidemic patterns can be tracked by watching the dynamic of search volume in related internet search activities for certain internet search queries. Internet search data enables investigators to discover disease patterns from timely intelligence at a larger spatial scale (18). As internet use worldwide is currently dominated by various search engines by country, reviewed studies used the dominating internet search engine data by study settings. In our review, several studies using Google (19, 20), Baidu (21–23), and other search term data (24–27) have been performed worldwide to successfully detect infectious disease events.

Social media communication is an increasingly utilized platform to monitor personal health information and contents, which is the main advancement of it compared to other internet data sources (28). Moreover, the interest in using social media data to track infectious diseases is increasing because of the timely data generated by internet users on the platform (29). Thus, social media data are a perfect source for detecting disease in the early stage because of their timeliness characteristic. This characteristic also enables health authorities to contact the public in the early phase of disease outbreak detection (30). We identified several original, exploratory studies on infectious diseases targeting social media users between 1 January 2000 and 30 June 2017. Both Twitter and other blogs claimed to be seen as valuable social media data sources in infectious disease surveillance (31–34).

### The data processing of internet search data

**Figure 1:**
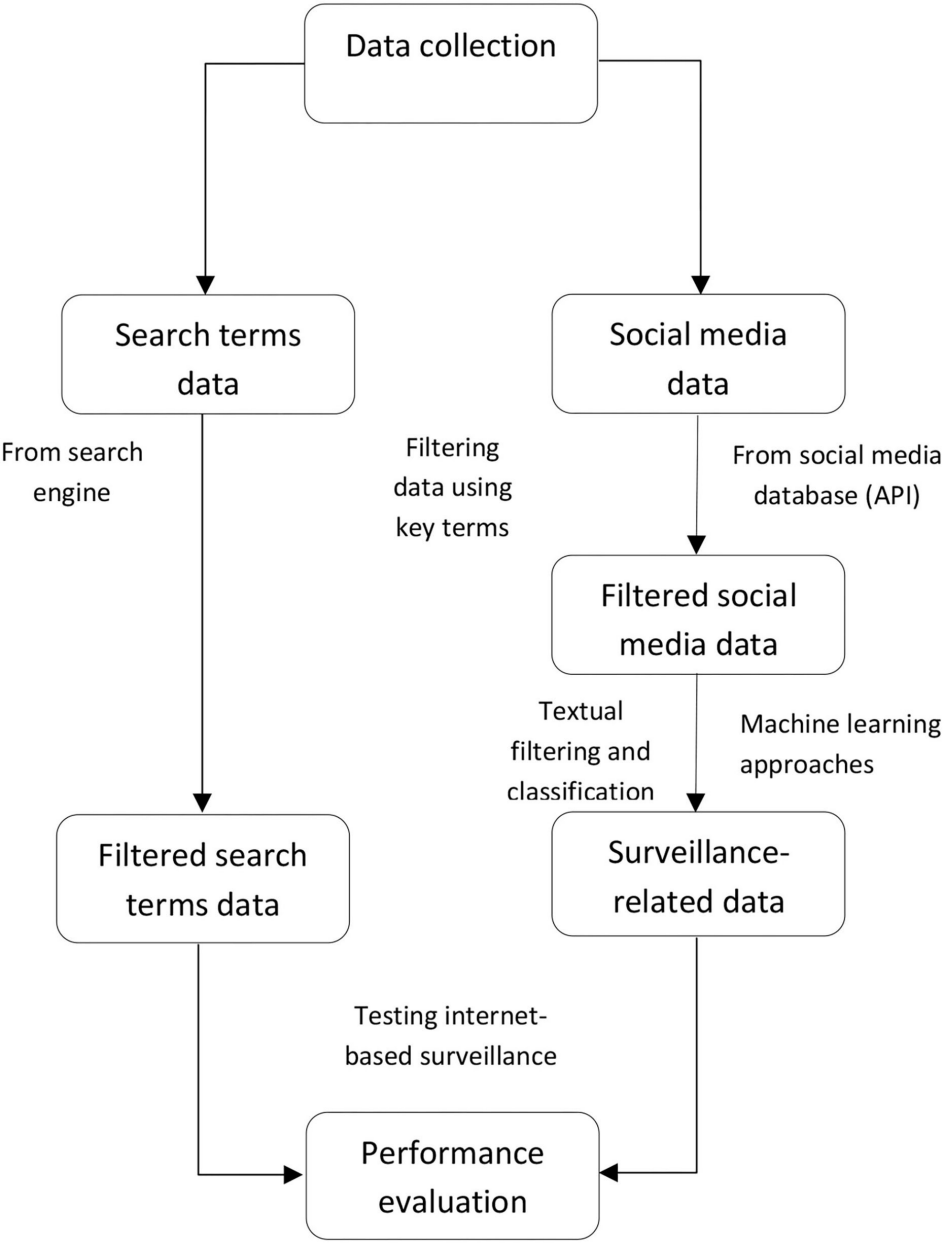
The flowchart of data collecting and processing for internet search data.

Through the common data collecting and processing steps of our reviewed studies (Figure 1), first, all data related to infectious diseases were collected from the internet. The studies that used “internet search data” as a variable collected the search volume from search engine websites and that used “social media data” as a data source that collected diseases-related contents through their application programming interface (API). In this stage, most of the included articles in this systematic review collected their data using key terms within specific time periods and locations. However, for social media data, not all data collected are associated with the specified diseases (35). Textual analysis of data was needed to identify disease-related and non-disease-related data to detect and track disease events. Thus, the second stage involves efficient social media data filtering and classification. Machine learning approaches are commonly performed in reviewed studies to classify whether the collected social media data are relevant to disease events (36). The final stage is to evaluate the predictive accuracy and time efficiency of internet-based surveillance compared to conventional surveillance.

## The spatiotemporal internet search data with analysis approaches

Traditional data analysis approaches often generate correlations with biases under the assumption that the independent variables have no autocorrelation at both spatial and temporal scales (37). However, such autocorrelation is very common in the real world.

The main advancement of internet search data is including rich spatiotemporal information, with uncertainty contents at both space and time scales. Internet search data generated from users' Internet Protocol Address can accurately reveal the users' locations. For example, internet search volume data (e.g., Google Trends) aggregated the internet search activities in a certain area with search terms. The geographically tagged tweets indicated the specific locations where the health issues occurred. This can avoid the spatial uncertainty between the real locations of the users and the geocoded address using coordinates, which has been widely used in traditional disease surveillance (38). Moreover, internet search data are produced in real time, including personal health issues, symptoms, and so on. Thus, using internet search data in disease surveillance can limit the time lag in the disease reporting process (39).

Spatiotemporal analysis in the domain of disease surveillance refers to analyzing the surveillance data with geospatial attributes in a time series (40). Elliott et al. identified several spatiotemporal analysis approaches in epidemiology, including disease mapping, disease cluster identification, and correlation analysis (41). Disease mapping is used to show the geographic location of events or attributes, which is useful for communicating trends or averages in an area. Moreover, disease cluster identification can ensure an unusually large aggregation of a relatively uncommon medical condition or event within a particular geographical location or period, which can be seen as an essential prerequisite for identifying an outbreak. Furthermore, the term “correlation analysis in disease surveillance” refers to measuring the strength of the association using statistical methods between event occurrence and potential risk factors, such as environmental factors and sociodemographic factors.

The advances in spatiotemporal analysis approaches can compensate for the residual variability as the spatial variation in data analysis processing, which may lead to a decrease in the effects of potential errors (42). It is critical to simultaneously include the spatiotemporal components and spatiotemporal uncertainty variables in the surveillance (43). Spatiotemporal analysis approaches have additional advances compared to the analysis methods that purely applied spatial or temporal analysis approaches, which is due to the dynamics in spatial patterns over time and temporal patterns at different spatial units. Overall, the spatiotemporal analysis approaches enable simultaneous data analysis at both space and time, as well as the investigation of any unusual spatiotemporal patterns (44).

### The spatiotemporal visualization in surveillance

As both internet search data and infectious disease surveillance data have rich information in space and time, an increasing number of studies used spatiotemporal visualization tools to map such variables. These tools enable researchers to show the spatiotemporal distribution of the route of disease transmission combined with the related internet search data. For instance, the HealthMap allows the demonstration of the map of currently active infectious diseases. Furthermore, the map contains the links for further latest information about the diseases, which were retrieved from the internet (45).

### The spatiotemporal clustering in surveillance

The detection of disease clusters plays a critical role in surveillance, which can help health organizations to identify relatively high-risk areas. Mackey and colleagues investigated the clusters of Tweets with COVID-19-related symptoms or experiences from March 3, 2020 to March 20, 2020. The results indicated that the regions with a larger number of population-normalized COVID-19 confirmed case exhibited more tweets with COVID-19 associated symptoms or experiences (46). Chowell et al. applied online news data and health bulletins to discover the clustering of Ebola virus disease (EVD) cases from January 2014 to January 2016. In the study, there was a high correlation coefficient (Spearman rho = 0.86; *P* < 0.001) between the monthly clusters number retrieved from online news and the officially reported number of EVD cases using traditional surveillance (47).

### The application of spatiotemporal models in surveillance

The high resolution of internet search data at both space and time has great promise to enhance disease surveillance by developing adaptive spatiotemporal models at different levels (national, state, and local government) of public health authorities (48).

Generous et al. applied internet search data on Wikipedia to forecast location of the disease in 14 countries with spatiotemporal linear models. Overall, the models successfully estimated the disease activities at a variety of time scales (49). Ma and Yang attempted to use Google Trends data to predict COVID-19 patterns in the United States at both national and state levels *via* regularized linear model, which incorporated a cross-state, cross-region spatiotemporal framework. The proposed model performed well in the predictions up to 4 weeks ahead (50). Zhang et al. developed seasonal auto-regressive integrated moving average (SARIMA) models in different local regions to discover the relationships between seasonal influenza epidemics and Google Trends data with identified key terms. The spatiotemporal contents were considered through the different parameters in the models by region, which can better fit the spatial heterogeneity (51). Li et al. developed generalized additive models (GAM) to predict dengue fever using Baidu Index data at the city level. The results indicated that internet search data (Baidu Index) promoted the forecasting performance at different time scales, compared to the model not using Baidu Index data (23).

The feature of internet search data provides a great opportunity to develop spatiotemporal models in surveillance at a finer spatial scale and time series. This enables public health authorities to better understand disease risks, especially in areas where traditional disease surveillance is poor (52). Furthermore, the flexible spatiotemporal modeling enables internet search data to generate dynamic surveillance in near real time (e.g., disease mapping and risk mapping) (53–56).

## The challenges in internet search data and spatiotemporal analysis

### The access to internet

As the internet search data is mainly based on the search activity online, it is crucial to consider the ability of internet access. A previous study reported that the majority of internet users were located in developing areas, such as Asia (53.7%), South America (10.2%), and Africa (10.1%) (57). As a result, these regions can be seen as the ideal places to collect internet search data. These regions may have great opportunities to develop infectious disease surveillance using internet search data.

### The internet search behavior

The overall performance of the models that purely used internet search data in infectious disease surveillance is widely noticed in previous research. However, such models may be subject to bias and come with potential errors. For instance, internet search data was applied to forecast the number of influenza peaking cases, but the number was two-fold higher than the number reported by the CDC (58). The accuracy of using internet search data in surveillance can be varied by internet search behaviors and media-driven bias. The widespread media reports may lead to many internet search activities by internet users who were not ill (59). Google Flu Trends (GFT) pioneered the internet search data-based flu surveillance in the world. GFT kept watching any changes in internet search behaviors to update its predictive models annually, which contribute to the goodness-of-fit to the reference flu surveillance data (60).

### The development of finer spatiotemporal analysis resolution

The main aim of developing infectious disease surveillance was to timely collect disease-related intelligence, which can contribute to decrease the impact of epidemics on the vulnerable population (32). Internet search data source is in an ideal location to conduct quick surveillance and monitoring as it can timely reflect epidemic patterns at the defined spatial units, where internet access is available (35). Internet search data usually included geographic information. Search engines usually provide search volume data at the state or even lower level, and social media data usually include users' geographic locations. This nature can identify high-risk areas of infectious diseases by determining the areas with high volumes of internet data generation.

Google Flu Trends have made some great progress in the finer spatiotemporal resolution analysis, which offers city-level or finer spatial resolution internet search data in influenza surveillance (61). Although a finer spatiotemporal resolution in internet search data is limited by the capacity of data aggregation and internet search volume, the rapidly increasing use of internet search as a health knowledge tool could lead to the development of a better spatiotemporal analysis using a finer resolution internet search data in space and time (62).

## Developing integrated surveillance using internet search data with spatiotemporal analysis

The surveillance system purely based on internet search data may generate noise value and failed predictive results. First, the accuracy of internet-based surveillance may be impacted by the levels of internet access (63). Second, it is acknowledged that there are different internet-seeking behaviors, self-reporting, and media-driven bias between different sectors of the community (64). Previous studies reported that media bias can adversely impact internet-based surveillance systems (65). Third, the absence of involving other risk factors for infectious diseases, such as climatic and socio-economic factors, may contribute to the noise or failed prediction of infectious disease events. Finally, exploring spatiotemporal clustering plays a crucial role in surveillance. This enables health authorities to trigger disease intervention in high-risk windows to reduce the burden and impact of infectious diseases.

The successful surveillance systems for infectious diseases are affected by the complex conditions and the transmission patterns of the disease, and social-environmental variables (66). Previous studies indicated that infectious diseases have strong climatic and social-environmental patterns (67, 68). The involvement of climatic and social-environmental factors could improve the predictive performance of epidemiological models (69–71). The prediction results can contribute to the decision-making of certain control measures and surveillance, such as the allocation of healthcare resources, social distance control, vaccination plan, and health education.

**Figure 2:**
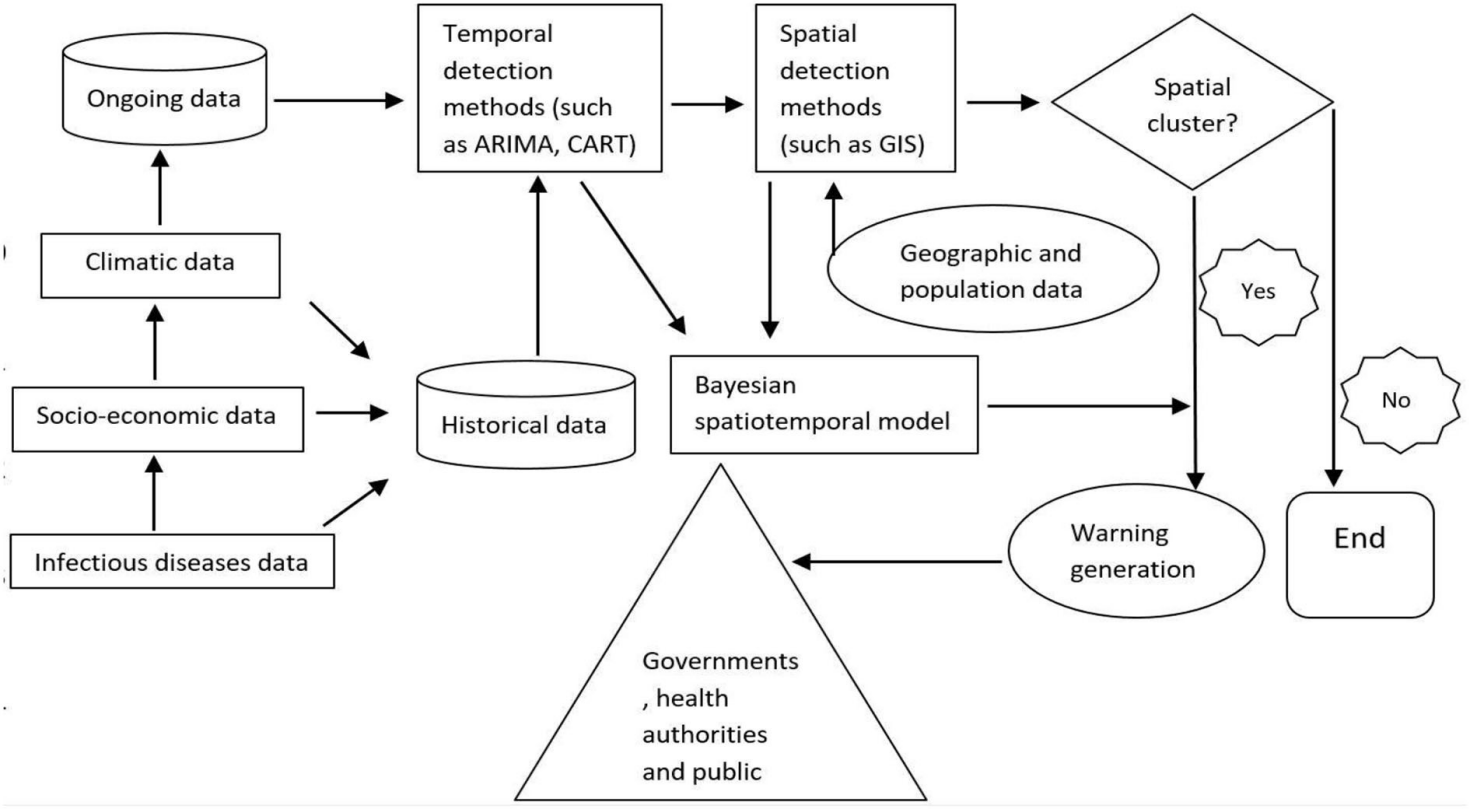
Hypothesized framework of a dynamic spatiotemporal model for infectious disease surveillance using internet search data.

Thus, a dynamic, integrated surveillance system using big data has the potential for timely and specifically detecting infectious disease events and reducing the potential errors introduced by factors such as fear-based searching. We designed a flowchart of a dynamic spatiotemporal model for infectious disease surveillance using big data, which provides possible research directions for future study (Figure 2).

## Conclusion

Internet search data hold the potential as a free, easily accessible data source to access large community fraction of health-related data to reflect disease activity and generate timely disease information by targeting people in the early phase of the disease process (72). Ongoing evaluation, validation, and verification of internet search data-based surveillance with epidemiological and clinical data by users, developers, and agencies will greatly improve the utilization of this new surveillance approach for infectious disease detection and tracking. This study provides the necessary groundwork and critical appraisal of the use of internet search data and spatiotemporal analysis approaches. This study also provides future directions to researchers to investigate the combination of internet search data with spatiotemporal analysis in a wider range of infectious disease surveillance in more regions worldwide.

## Data availability statement

The original contributions presented in the study are included in the article/supplementary material, further inquiries can be directed to the corresponding author.

## Author contributions

HS and YZ contributed to the conceptualization, methodology, and writing—review and editing. GG and DW contributed to the data curation and funding acquisition. All authors read and agreed to the published version of the manuscript, participated in data collection, preliminary analysis, early drafting of the manuscript, made substantive contributions to the development and revision of the manuscript, contributed to the article, and approved the submitted version.

## Funding

This research was supported by the Major Scientific and Technological Projects in Ningbo (2021Z050) and the Science and Technology Project of Zhejiang Provincial Department of Natural Resources (2020-16).

## Conflict of interest

HS, YZ, GG, and DW were employed by Popsmart Technology (Zhejiang) Co., Ltd.

## Publisher's note

All claims expressed in this article are solely those of the authors and do not necessarily represent those of their affiliated organizations, or those of the publisher, the editors and the reviewers. Any product that may be evaluated in this article, or claim that may be made by its manufacturer, is not guaranteed or endorsed by the publisher.
